# Impact of cardiac cycle and respiratory rhythm phase on visual attention in healthy young and older adults

**DOI:** 10.1038/s41598-026-47916-6

**Published:** 2026-04-18

**Authors:** Praghajieeth Raajhen Santhana Gopalan, Jarmo Hämäläinen, Markku Penttonen, Miriam S. Nokia

**Affiliations:** Department of Psychology and Centre for Interdisciplinary Brain Research, University of Jyväskylä, Jyväskylä, Finland

**Keywords:** Alerting, Executive control, Aging, Cardiac cycle, Breathing, Electroencephalogram, Neuroscience, Physiology, Psychology, Psychology

## Abstract

Visual attention is crucial for human cognition, and previous research suggests that cardiorespiratory rhythms may modulate attentional processing. We investigated how cardiac and respiratory cycle phases influence visual attentional subprocesses: alerting and conflict-related processing. Healthy young and older adults (*n* = 25 per group) performed the Attention Network Test (ANT) while electroencephalography, respiration, and electrocardiography were recorded. We expected shorter reaction times (RTs) and larger event-related potential (ERP) amplitudes during inspiration and diastole compared to expiration and systole, respectively, with stronger effects in young adults. Behaviorally, young adults showed larger cue-related benefits than older adults, whereas conflict effects did not differ between groups. No reliable effects of cardiac or respiratory phase were observed on RTs. In contrast, ERP amplitudes revealed physiological modulation: respiratory phase produced task-dependent effects on N1 and P3 amplitudes, whereas cardiac phase exerted a robust global modulation, with larger amplitudes during late diastole compared to systole. These physiological effects did not differ between age groups, indicating preserved cardiorespiratory modulation of neural attentional processing in healthy aging. Future research should explore how individual differences in cardiorespiratory fitness and brain–body rhythm synchronization influence attention and learning.

## Introduction

Visual attention can be divided into three key subprocesses: alerting, orienting, and executive control. Alerting refers to achieving and maintaining a state of high sensitivity to incoming stimuli, orienting involves directing attention to specific spatial locations, and executive control involves managing conflict between competing stimuli or responses^1^. Beyond this classical framework, accumulating evidence suggests that visual attention can be modulated by internal physiological rhythms, particularly those of the respiratory and cardiac systems.

Regarding respiration, results from a multitude of behavioral and neurophysiological studies suggest that inspiration facilitates perceptual and cognitive processing^2–6^. In a visual object memory task and an emotional face discrimination task, healthy young adults reacted faster and more accurately when stimuli were presented during natural nasal inspiration compared to expiration^3^. Supporting these findings, in a visuospatial cueing task, inspiration at trial onset was associated with improved visuospatial task performance and enhanced parieto-occipital N2 event-related potential (ERP) amplitudes^4^. In a near-threshold visual detection task, electroencephalography (EEG) data further showed that inspiration increased subjective perceptual awareness and sped up decision making without compromising accuracy^5^. Similarly, in a visual threshold task using backward-masked stimuli, healthy young adults had enhanced early ERP components (P1), during inspiration, interpreted to reflect improved early visual processing^6^. Magnetoencephalography (MEG) studies have also produced results supporting the idea of a respiratory-phase effect: in a rapid visual detection paradigm, perceptual thresholds were lower during inspiration than expiration^2^. Collectively, these findings suggest that inspiration may synchronize neural activity to optimize attentional and perceptual readiness.

However, there are also a number of studies that have produced results conflicting with the above view. Some visual memory studies report no respiratory-phase effects on performance at all^7–9^ while others suggest that a different phase of respiration might benefit cognition: in a delayed recognition task, recall accuracy was reduced when the retrieval phase included the transition from expiration to inspiration^10^. In addition, self-paced paradigms where stimulus onset is determined internally rather than externally have shown no consistent respiratory-phase effects^11^. Such inconsistencies raise questions about generalizability and the role of task structure and cognitive demands.

Beyond respiration, the cardiac cycle has also been shown to influence visual attention, possibly through baroreceptor-mediated effects on sensory processing. A recent review of cardiac-phase effects across perceptual and motor domains reported that systole typically suppresses sensory processing due to increased baroreceptor activity, while diastole facilitates perception, particularly in tasks with low stimulus salience^12^. In a dynamic visual search task, reaction times (RTs) to targets were slower when stimuli appeared during systole (linked to baroreceptor noise) compared to diastole, though accuracy remained unaffected^13^. In a stop-signal task, diastole was associated with larger P3 amplitudes, which have been interpreted as reflecting modulation of task-related stimulus evaluation and cognitive control processes during action stopping^14^. Further supporting this pattern: saccadic eye movements occurred more frequently during systole, while fixations, which help maintain stable visual attention, were linked to diastole^15^. However, in a face detection task using emotional stimuli, faster responses were observed during systole, suggesting that emotional salience may reverse the typical cardiac-phase effect^16^. In a near-threshold tactile detection task, detection rates were higher during early expiration and diastole, and participants showed spontaneous respiratory phase-locking to stimulus timing, suggesting that bodily rhythms may synchronize with perceptual expectations^17^. Overall, these studies indicate that diastole generally supports visual attention, although cardiac-cycle influences appear to depend on task demands and stimulus characteristics.

Previous research has linked respiratory and cardiac phases to changes in behavioral performance and ERP components such as the N2 and P3 in adults. However, their influence on early visual attention (as reflected by the N1) and conflict-related processing (as indexed by the P3) remains unclear. Further, bodily rhythm effects on attention have not been studied in older individuals while it is known that aging is generally associated with slower RTs and diminished benefits from alerting cues^18–20^. Previously, attenuated posterior N1 responses following alerting cues have been reported in older adults, indicating reduced cortical responsiveness to preparatory signals^18^. Findings on the effects of aging on executive control or conflict processing are mixed: Some studies have reported larger flanker interference effects and reduced P3 amplitudes in older adults^19^, while others suggest that differences in executive control may only emerge in very old age or in individuals at risk for cognitive decline^20^. These findings collectively indicate that aging selectively impairs certain attentional subprocesses, particularly alerting and executive control. However, the interaction between physiological rhythms and these attentional networks in aging remains poorly understood and warrants further investigation.

In this study, we aim to investigate how phases of respiration (i.e., inspiration and expiration) and cardiac activity (i.e., systole and diastole) influence RTs and visual ERP components related to alerting (N1) and conflict-related processing (P3) in healthy young and older adults. We use a modified version of the visual Attention Network Test (ANT)^21^. We hypothesize that inspiration and diastole will be associated with faster RTs and enhanced N1 and P3 amplitude compared to expiration and systole, respectively^4,13,14,22^. We will also compare RTs and attention-related ERP components between young and older participants. We expect faster RTs and increased alerting in young compared to older adults^18,23^. Additionally, we anticipate reduced efficiency of executive control in older adults compared to young adults. These age-related differences may be attributed to changes in cognitive processing and attention regulation mechanisms across the lifespan. With this study, we aim to provide a comprehensive understanding of how cardiorespiratory rhythms influence attentional subprocesses and how age-related changes impact these dynamics.

## Results

**Fig. 1:**
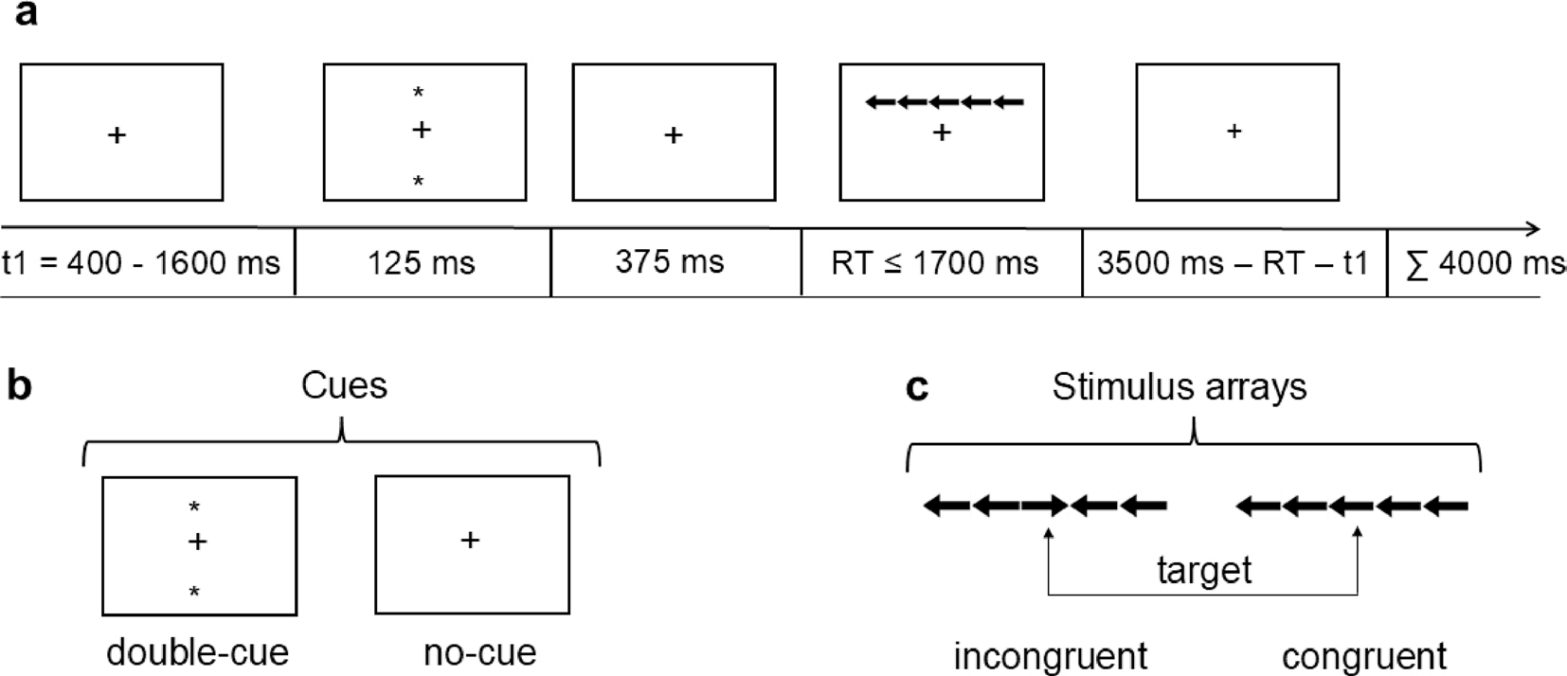
Attention Network Test (ANT) design. Schematic illustrations of (**a**) the sequence of events in the ANT, (**b**) the two cue conditions used in ANT, and (**c**) the two target stimulus conditions for which the participant had to decide the direction of the middle arrow (target). A fixation cross remained continuously visible throughout the entire trial. Each trial began with either no cue or a double cue, followed by an array of arrows. Participants indicated the direction of the central target arrow as quickly and accurately as possible, using their dominant hand to press a button.

Participants performed a modified version of the visual Attention Network Test (ANT), which included two cue conditions (no-cue and double-cue) and two flanker conditions (congruent and incongruent). These conditions allowed us to quantify alerting effects (double-cue vs. no-cue) and conflict-related effects (incongruent vs. congruent) at both the behavioral and electrophysiological levels. A schematic overview of the task design and trial structure is shown in Fig. 1.

### The alerting effect on reaction times was larger in young adults than in older adults, while there was no difference in the conflict effect

All participants maintained a high level of accuracy throughout the ANT. Mean accuracy (%) was 98.6 and 97.6 for double-cue, 98.3 and 97.6 for no-cue, 97.4 and 96.0 for incongruent, and 99.6 and 99.2 for congruent trials in young and older adults, respectively. The mean and standard deviation of reaction times (RTs) for double-cue target stimuli were 602 ± 67 ms for young adults and 811 ± 103 ms for older adults. RTs for no-cue target stimuli were 649 ± 76 ms for young adults and 843 ± 99 ms for older adults. RTs for incongruent stimuli were 692 ± 79 ms for young adults and 920 ± 123 ms for older adults. RTs for congruent target stimuli were 560 ± 67 ms for young adults and 739 ± 92 ms for older adults.

A mixed-design ANOVA with Cue (double, no-cue) and Conflict (congruent, incongruent) as within-subject factors and Age group (young, older) as a between-subject factor revealed a significant three-way interaction, Cue × Conflict × Age group, F [1, 47] = 6.62, *p* = 0.013, η² = 0.12. To contextualize this interaction, the overall model showed robust main effects of Cue, F [1, 47] = 190.59, *p* < 0.001, η² = 0.80, and Conflict, F [1, 47] = 526.18, *p* < 0.001, η² = 0.92, and a clear Age group effect, F [1, 47] = 62.73, *p* < 0.001, η² = 0.57, with older adults responding more slowly overall. There were also significant two-way interactions of Cue × Conflict, F [1, 47] = 18.93, *p* < 0.001, η² = 0.29, Cue × Age group, F [1, 47] = 8.30, *p* = 0.006, η² = 0.15, and Conflict × Age group, F [1, 47] = 12.43, *p* < 0.001, η² = 0.21.

**Fig. 2:**
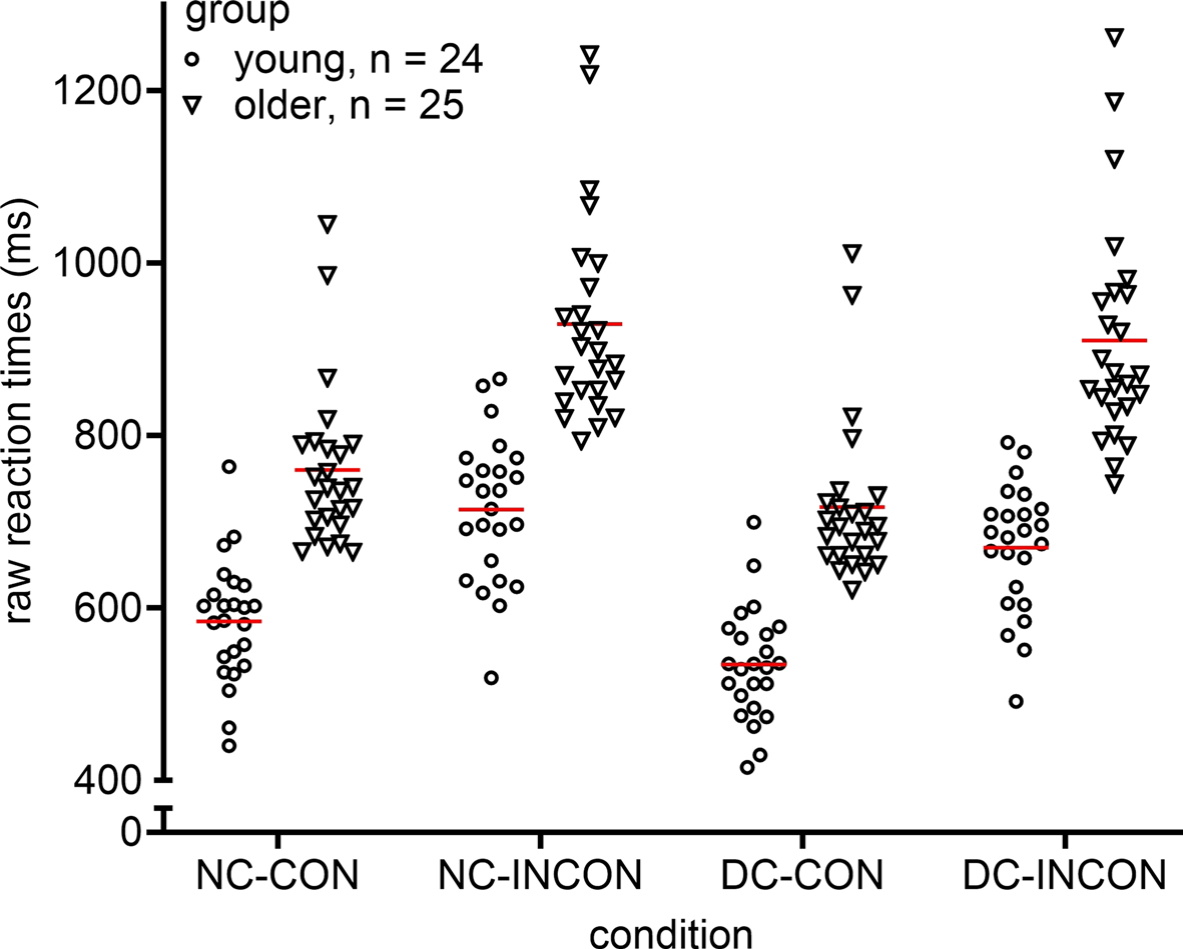
Cueing reduced reaction times more for congruent than for incongruent trials in older adults, whereas cueing and congruency effects were similar in young adults. Raw reaction times are shown for no-cue congruent (NC–CON), no-cue incongruent (NC–INCON), double-cue congruent (DC–CON), and double-cue incongruent (DC–INCON) trials in young and older adults.

Because the three-way Cue × Conflict × Age interaction was significant, we conducted follow-up analyses separately for each age group. In young adults, both Cue and Conflict showed strong main effects (Cue: F [1, 23] = 183.01, *p* < 0.001, η² = 0.89; Conflict: F [1, 23] = 341.77, *p* < 0.001, η² = 0.94), but their interaction was not significant, F [1, 23] = 2.03, *p* = 0.168, η² = 0.08. That is, RTs were slower during uncued than cued trials and slower during incongruent than congruent trials. In older adults, Cue and Conflict also showed robust main effects (Cue: F [1, 24] = 48.97, *p* < 0.001, η² = 0.67; Conflict: F [1, 24] = 248.15, *p* < 0.001, η² = 0.91), and the Cue × Conflict interaction was significant, F [1, 24] = 19.97, *p* < 0.001, η² = 0.45. To clarify this interaction, we examined simple effects in older adults. Older adults showed a significant cueing benefit for congruent trials [t (24) = 8.65, *p* < 0.001, Cohen’s d = 1.73]. A smaller but still significant cueing benefit was found for incongruent trials [t (24) = 3.61, *p* = 0.001, Cohen’s d = 0.72]. Thus, the three-way interaction reflects that cueing and conflict effects are independent in young adults but interact in older adults (See Fig. 2).

**Fig. 3:**
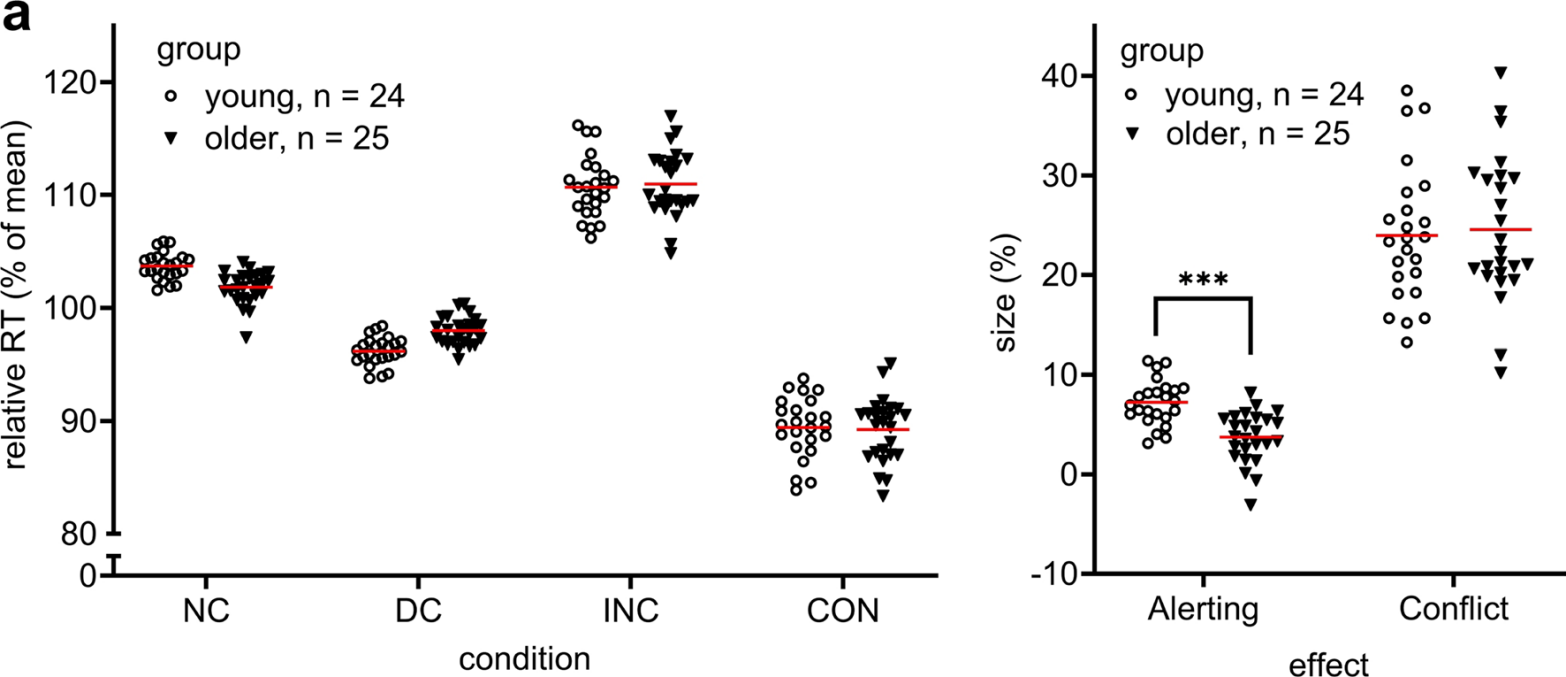
Effects of cue condition (no-cue, NC vs. double-cue, DC) and flanker condition (incongruent, INC vs. congruent, CON) on reaction time (RT). The alerting effect was larger in young adults than in older adults. (**a**) Relative RTs (% of each participant’s mean) during the different Attention Network Test conditions illustrate the alerting effect and conflict effect: RTs are slower for no-cue targets compared to double-cue targets and slower for incongruent vs. congruent targets. (**b**) The size (%) of the alerting effect [(NC - DC) / NC * 100] was larger in young compared to older adults (t-test, *p* < 0.001) but there was no difference between groups in the size of the congruency effect [(INC - CON) / CON * 100].

To control for general age-related slowing, RTs were normalized to each participant’s mean (% of the mean). Relative RTs during each trial type are depicted in Fig. 3a, and the size (%) of the alerting and conflict effects is shown in Fig. 3b. The normalized RT data were analyzed using the same mixed-design ANOVA with Cue (double, no-cue) and Conflict (congruent, incongruent) as within-subject factors and Age group (young, older) as a between-subject factor. This analysis revealed significant main effects of Cue, F [1, 47] = 254.83, *p* < 0.001, η² = 0.84, and Conflict, F [1, 47] = 724.50, *p* < 0.001, η² = 0.94, as well as significant Cue × Conflict, F [1, 47] = 16.74, *p* < 0.001, η² = 0.26, and Cue × Age group interactions, F [1, 47] = 26.77, *p* < 0.001, η² = 0.36. The three-way Cue × Conflict× Age group interaction did not reach statistical significance, F [1, 47] = 3.51, *p* = 0.067, η² = 0.07. Thus, while the pattern of normalized RTs qualitatively mirrored that observed for raw RTs, including a reduced alerting effect in older adults, the interaction between cueing and congruency did not differ significantly between the age groups after normalization. Overall, these results indicate that age-related differences in alerting efficiency persist after controlling for general slowing, whereas conflict effects remain comparable between young and older adults.

### Effects of respiration phase and cardiac cycle on reaction times

**Fig. 4:**
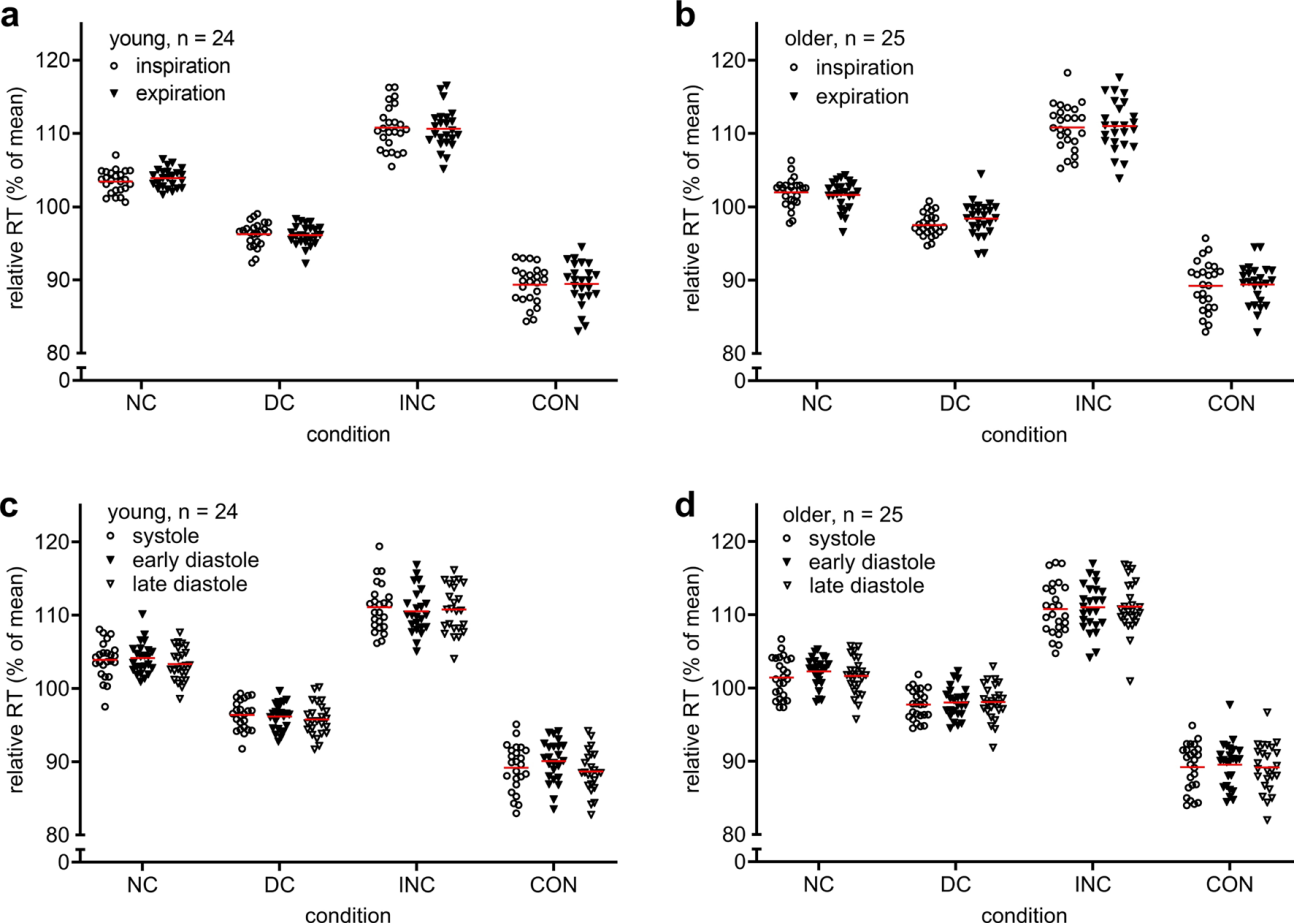
The effect of respiratory and cardiac cycle phases on reaction time (RT) during the Attention Network Test. (**a**) RTs during inspiration vs. expiration in young adults and (**b**) in older adults. (**c**) RTs during systole, early diastole and late diastole in young adults and (**d**) in older adults. The four conditions are: no-cue (NC), double-cue (DC), incongruent (INC), and congruent (CON). Note that RTs did not differ between inspiration and expiration or between the phases of the cardiac cycle.

The relative RTs for all ANT conditions during inspiration and expiration are illustrated in Fig. 4a (young adults) and Fig. 4b (older adults). To assess whether respiration phase modulated RTs differently across task conditions or age groups, relative RTs were analyzed using a mixed-design repeated-measures ANOVA with Phase (inspiration, expiration) and Condition as within-subject factors and Age group as a between-subject factor. This analysis revealed no significant main effect of Phase, F [1, 47] = 0.84, *p* = 0.364, η² = 0.018, and no Phase × Age group interaction, F [1, 47] = 1.27, *p* = 0.266, η² = 0.026. The Phase × Condition interaction was also not significant, F [3, 141] = 1.05, *p* = 0.373, η² = 0.022, nor was the Phase × Condition × Age group interaction, F [3, 141] = 0.91, *p* = 0.438, η² = 0.019. Thus, the respiration phase did not significantly influence reaction times, nor did it interact with task condition or age group.

**Fig. 5:**
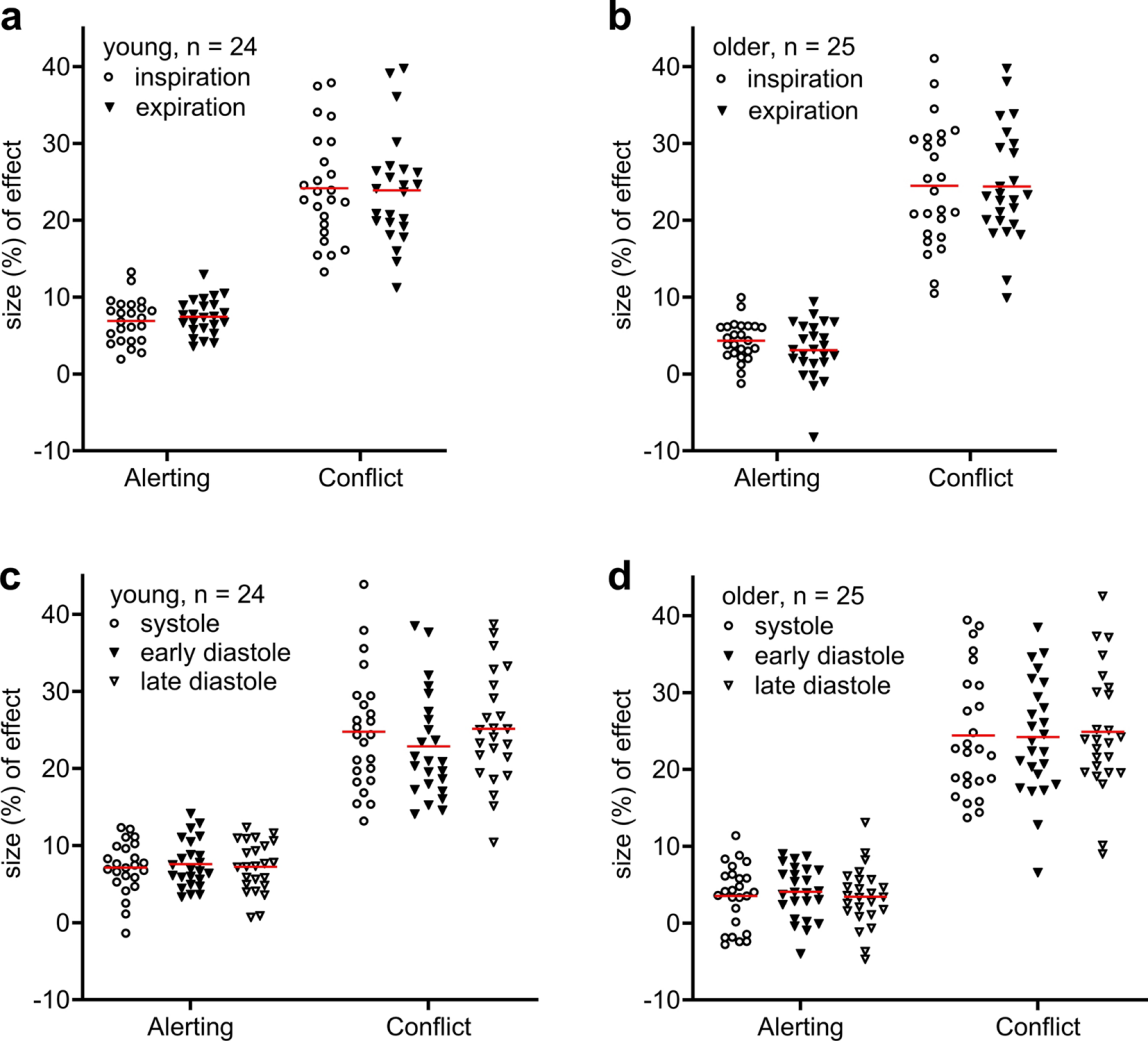
The effect of respiratory and cardiac cycle phase on the alerting and conflict effects quantified as the difference (%) in reaction time to the target between the different stimulus conditions in the Attention Network Test. (**a**) Alerting and **conflict** effect during inspiration vs. expiration in young adults and (**b**) in older adults. (**c**) Alerting and **conflict** effect (%) during cardiac cycle phases in young adults and in (**d**) older adults.

The relative RTs across the three phases of the cardiac cycle (systole, early diastole, late diastole) for all ANT conditions are illustrated in Fig. 4c (young adults) and Fig. 4d (older adults). A mixed-design repeated-measures ANOVA with Cardiac phase and Condition as within-subject factors and Age group as a between-subject factor revealed no significant main effect of Cardiac phase, F [2, 94] = 1.08, *p* = 0.344, η² = 0.022, and no Cardiac phase × Age group interaction, F [2, 94] = 1.27, *p* = 0.286, η² = 0.026. While a robust main effect of Condition was observed, F [3, 141] = 96.42, *p* < 0.001, η² = 0.67, neither the Cardiac phase × Condition interaction, F [6, 282] = 1.31, *p* = 0.248, η² = 0.027, nor the Cardiac phase × Condition × Age group interaction reached significance, F [6, 282] = 1.19, *p* = 0.310, η² = 0.025. The condition-specific RT patterns across cardiac phases are further illustrated in Fig. 5 for descriptive purposes only.

Finally, alerting and conflict effects were analyzed using mixed-design repeated-measures ANOVAs. No significant effects of respiration phase or cardiac cycle were observed on either alerting or conflict effect, and no interactions with Age group were found (all F ≤ 2.13, all *p* ≥ 0.09, all η² ≤ 0.11). Together, these findings indicate little to no systematic influence of respiration phase or cardiac cycle on reaction times or attentional effects in the ANT. Although isolated within-condition comparisons suggested small descriptive differences in some cases, these did not correspond to significant interaction effects and were therefore not interpreted as reliable condition- or group-specific phase influences.

### Cardiac phase, but not respiration phase, modulates N1 and P3 amplitudes

**Fig. 6:**
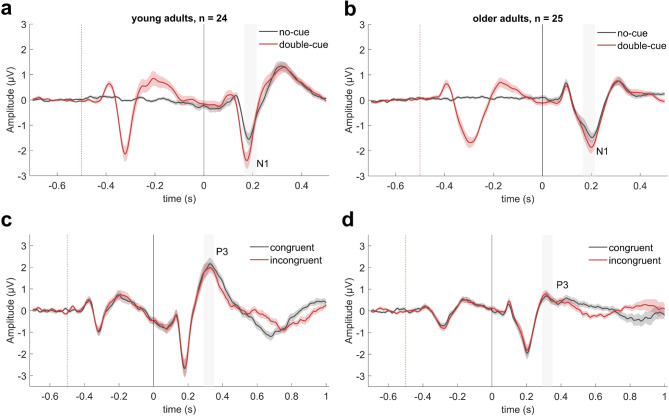
Grand-averaged visual event-related potential waveforms from selected electrodes recorded during the Attention Network Test illustrate N1 and P3 response to the target in young and older adults. The waveforms show the group mean ± SEM, with SEM as the light-colored shading. Grey shading indicates the N1 and P3 time windows. (**a**) N1 in young adults. (**b**) N1 in older adults. (**c**) P3 in young adults. (**d**) P3 in older adults. A and B show the mean of electrodes from Oz, O1, O2, PO7, PO8, POz, P3, and P4. C and D show the mean of electrodes from Pz, POz, and Oz. The target onset is marked at 0 s (black vertical line, all trials) and the possible cue onset is marked at -500 ms (red vertical dashed line, half the trials). The four stimulus conditions are: no-cue, double-cue, congruent, and incongruent.

N1 and P3 amplitudes were analysed across all ANT conditions to examine global phase-related effects. For respiration phase (inspiration vs. expiration), no significant main effects were observed on either N1 or P3 amplitudes. For the N1, the main effect of respiration phase was not significant, F [1, 47] = 0.001, *p* = 0.980, η² = 0.000, and the respiration phase × age group interaction was also not significant, F [1, 47] = 0.101, *p* = 0.752, η² = 0.002. Similarly, for the P3 component, there was no significant main effect of respiration phase, F [1, 47] = 0.135, *p* = 0.715, η² = 0.003, and no respiration phase × age group interaction, F [1, 47] = 0.041, *p* = 0.840, η² = 0.001. Thus, when averaged across task conditions, respiration phase did not systematically modulate N1 or P3 amplitudes in either age group (Fig. 6).

In contrast, cardiac cycle phase (systole, early diastole, late diastole) significantly modulated ERP amplitudes. For the N1 component, a significant main effect of cardiac phase was observed, F [1.71, 80.25] = 6.23, *p* = 0.005, η² = 0.117 (Greenhouse–Geisser corrected). The cardiac phase × age group interaction was not significant, F [1.71, 80.25] = 0.33, *p* = 0.688, η² = 0.007, indicating comparable modulation across age groups. Follow-up within-subject contrasts revealed a significant linear increase in N1 amplitude from systole to late diastole, F [1, 47] = 9.37, *p* = 0.004, η² = 0.166, whereas the quadratic trend was not significant (*p* > 0.10).

For the P3 component, cardiac phase also showed a significant main effect, F [1.49, 70.13] = 11.66, *p* < 0.001, η² = 0.199 (Greenhouse–Geisser corrected). The cardiac phase × age group interaction did not reach statistical significance, F [1.49, 70.13] = 3.03, *p* = 0.069, η² = 0.061. Within-subject contrasts indicated a significant linear increase in P3 amplitude from systole to late diastole, F [1, 47] = 17.85, *p* < 0.001, η² = 0.275, whereas the quadratic trend was not significant (*p* > 0.10).

In summary, when collapsed across task conditions, respiration phase did not exert a global influence on N1 or P3 amplitudes, whereas cardiac cycle phase significantly modulated both ERP components in young and older adults, with largely age-independent effects.

### Visual event-related potential components related to alerting (N1) and conflict-related processing (P3) differ between young and older adults

**Fig. 7:**
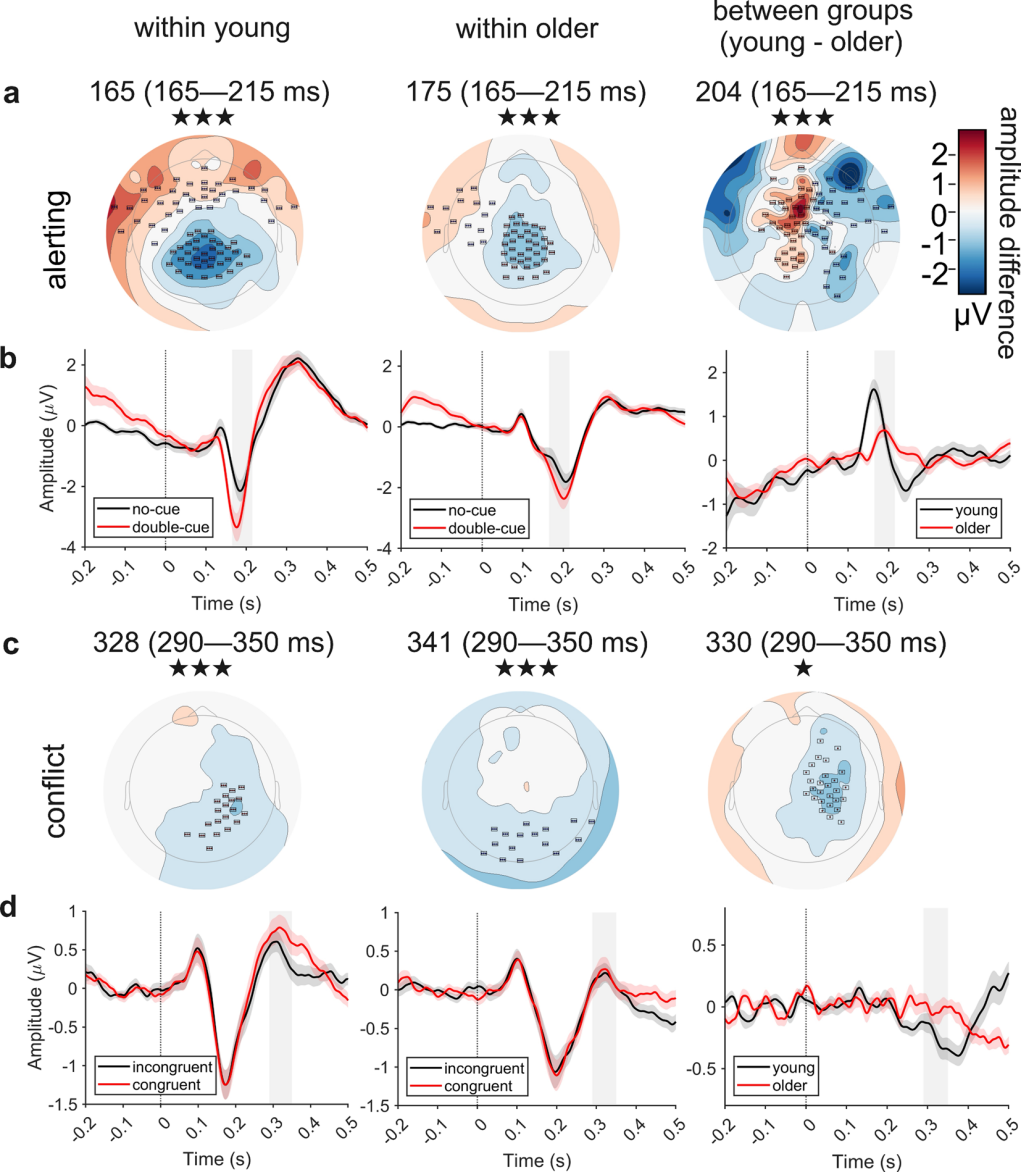
Visual target-evoked event-related potential (ERP) N1 and P3 amplitude differences (µV) between stimulus conditions in the Attention Network Test indicate clear alerting and conflict effects, respectively. Both young and older adults showed larger N1 and P3 amplitudes when a double cue or congruent flankers were present, compared with no-cue and incongruent stimuli. Young adults exhibited larger N1 and P3 responses than older adults under these conditions. (**a**) Topographical maps showing differences in N1 amplitude related to the alerting effect (double-cue minus no-cue) in young (left) and older (middle) adults and their between-group difference (young minus older, right). (**b**) Grand-average ERP waveforms extracted from cluster-derived posterior regions of interest (ROIs; electrodes 71, 72, 75, 76, and 77) for the alerting effect, shown in the same order as in (a). ERP waveforms were baseline corrected using the − 200 to 0 ms pre-cue interval during preprocessing and are shown here time-locked to target onset to highlight target-related responses. (**c**) Topographical maps showing differences in P3 amplitude related to the conflict effect (incongruent minus congruent), displayed in the same order as in (a). (**d**) Grand-average ERP waveforms extracted from cluster-derived centroparietal ROIs electrodes 92, 93, 94, 95, 96, and 97 for the conflict effect, shown in the same order as in (c). Vertical dashed lines indicate that 0 s corresponds to target onset. Shaded grey areas indicate the significant cluster time window. Statistical significance was assessed using cluster-based permutation tests (two-tailed t-tests). Electrodes belonging to significant clusters are indicated by black square markers, and stars denote the significance level of the cluster. The numbers above each plot represent the latency (ms) from target onset at which the maximum t-value occurs within the significant cluster (cluster time window shown in parentheses). Color bars represent ERP amplitude differences (µV). The rightmost topographies (“Between Groups”) depict between-group statistical comparisons of the difference waveforms (Young − Older). Significance levels are denoted as follows: ★ *p* < 0.050 and ★★★ *p* < 0.001.

Results of the cluster-based permutation tests are illustrated in Fig. 7. Paired-samples t-tests revealed statistically significant alerting effects (i.e., double-cue vs. no-cue; Fig. 7a-b) and conflict effects (i.e., incongruent vs. congruent; Fig. 7c-d) in both young and older participants.

A significant N1 amplitude difference was found in both young and older adults within 165–215 ms from target onset (*p* < 0.0001). In both age groups, larger N1 amplitudes were observed over bilateral occipito-parietal electrodes for target stimuli preceded by a double-cue compared to target stimuli preceded by no-cue. In young adults, and in the between-group comparison, an additional significant cluster with opposite polarity and a more anterior scalp distribution were observed (Fig. 7a), whereas in older adults the alerting effect was primarily characterized by the posterior N1 cluster. Significant differences in the alerting effect were found between young and older adults within the same latency window (165–215 ms; *p* < 0.0001), with young adults showing larger effects than older adults. This between-group contrast was characterized by two significant clusters with opposite polarity within this latency window.

Significant P3 amplitude differences between incongruent and congruent trials reflecting conflict-related processing were observed within 290–350 ms from target onset in both young (*p* = 0.001) and older adults (*p* < 0.0001). In both age groups, larger P3 amplitudes were observed over centro-parietal and occipital electrodes for target stimuli presented among congruent flankers compared to target stimuli presented among incongruent flankers. Significant differences in the conflict effect were found between young and older adults within the same latency window (290–350 ms; *p* = 0.009), with young adults showing larger effects than older adults.

In summary, both young and older adults showed larger N1 and P3 amplitudes for double-cue and congruent target stimuli compared to no-cue and incongruent stimuli, respectively. Additionally, young adults exhibited larger task-related brain responses than older adults.

### The N1 and P3 evoked by the visual target during ANT differ in amplitude between inspiration and expiration

**Fig. 8:**
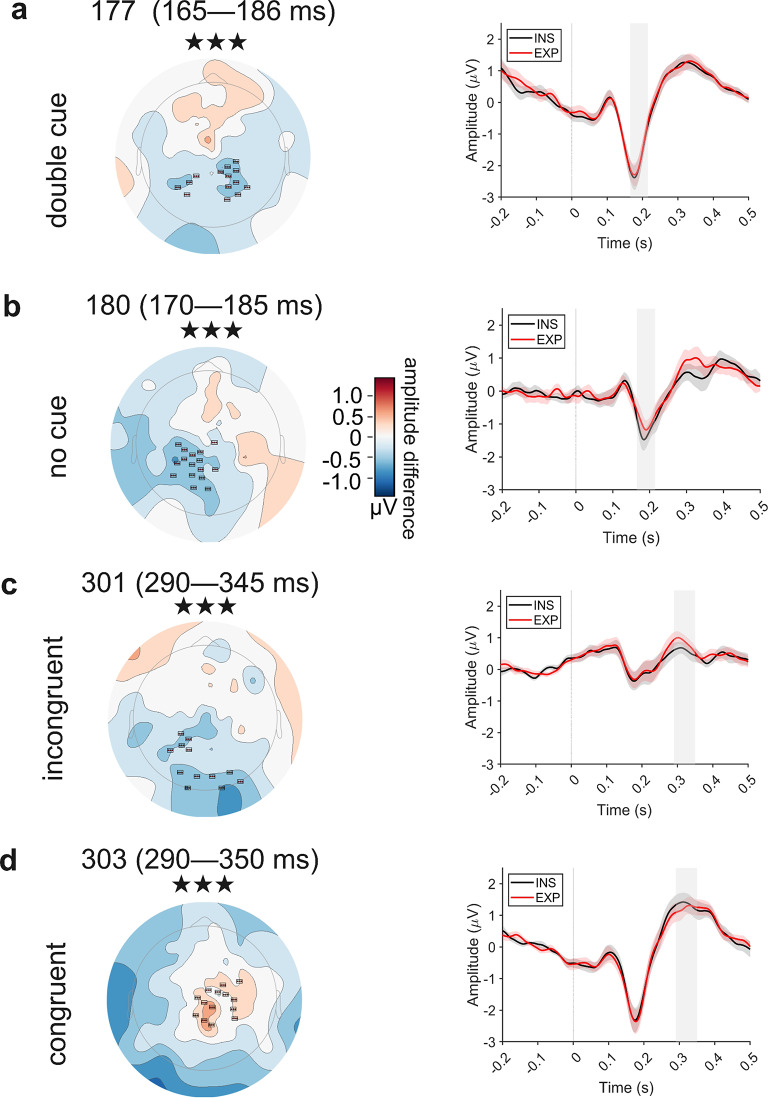
Topographical maps and event-related potentials (ERPs) showing the effects of respiratory phase (inspiration vs. expiration) on visual target-evoked N1 (165–215 ms) and P3 (290–350 ms) amplitudes during the Attention Network Test in young adults. Rows correspond to stimulus conditions as follows: (**a**) double-cue, (**b**) no-cue, (**c**) incongruent, and (**d**) congruent. For each condition, scalp topographies (left panels) display the amplitude differences (µV) between inspiration and expiration separately for young adults. Electrodes belonging to significant clusters identified using cluster-based permutation tests (two-tailed t-tests) are marked by black squares, and stars indicate the significance level of the clusters (★★ *p* < 0.010, ★★★ *p* < 0.001). Numbers above each topography indicate the latency (ms) of the peak t-value within the significant cluster, with the cluster time window shown in parentheses. Grand-average ERPs averaged across electrodes belonging to the significant clusters are shown in the right panel (black = inspiration, red = expiration). Vertical dashed lines indicate that 0 s corresponds to target onset. Vertical shaded grey areas indicate the significant cluster time window.

Results for young adults are illustrated in Fig. 8. Cluster-based permutation tests revealed that during the double-cue condition (Fig. 8a), young adults had larger N1 amplitude during inspiration than expiration over parieto-occipital areas within 165–186 ms (*p* < 0.0001) from the target onset. Although the spatial extent of the N1 cluster appeared slightly asymmetric in the young adults in the double-cue condition (Fig. 8), a direct left–right comparison of the cluster electrodes showed no significant lateralisation (left vs. right: t(23) = − 0.48, *p* = 0.63, Cohen’s dz = − 0.10). For no-cue stimuli (Fig. 8b), young adults showed larger N1 amplitudes during inspiration than expiration over left parieto-occipital areas within 170–185 ms (*p* < 0.0001) of the target onset. For incongruent stimuli (Fig. 8c), young adults had a larger P3 amplitude during expiration than inspiration in the left occipital areas within 290–345 ms (*p* < 0.0001) from target onset. For congruent stimuli (Fig. 8d), young adults showed larger P3 amplitudes during inspiration compared to expiration in the centro-parietal and occipital areas within 290–350 ms (*p* = 0.001).

**Fig. 9:**
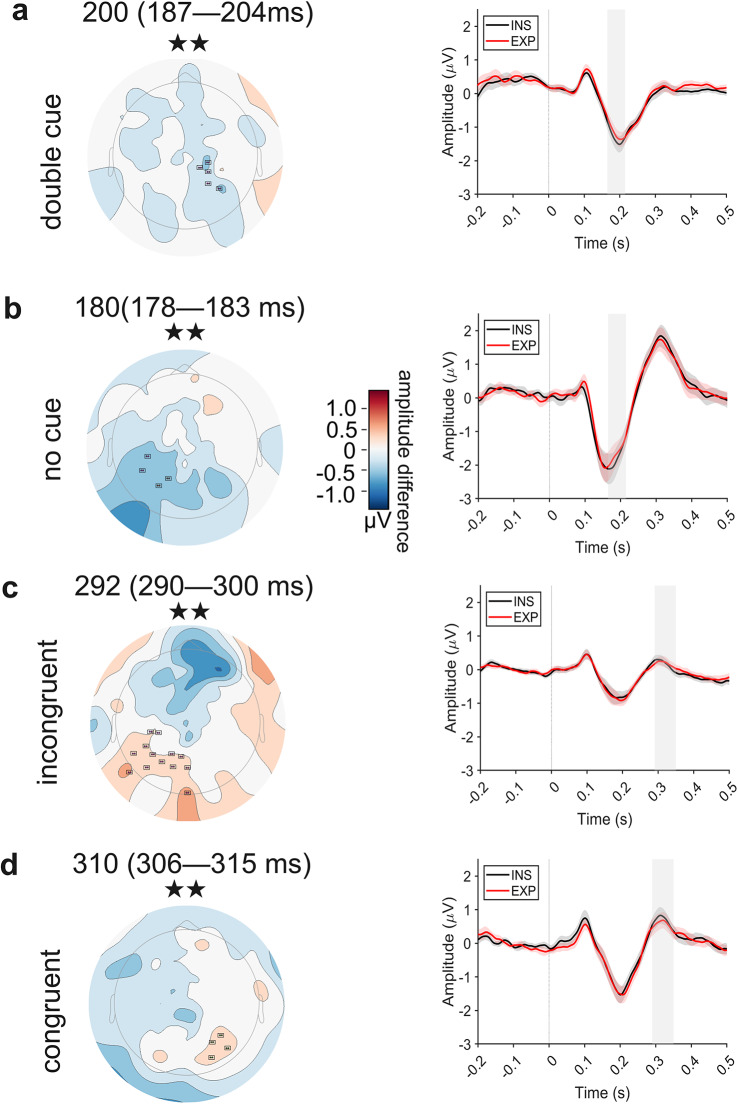
Topographical maps and event-related potentials (ERPs) showing the effects of respiratory phase (inspiration vs. expiration) on visual target-evoked N1 (165–215 ms) and P3 (290–350 ms) amplitudes during the Attention Network Test in older adults. Rows correspond to stimulus conditions as follows: (**a**) double-cue, (**b**) no-cue, (**c**) incongruent, and (**d**) congruent. For each condition, scalp topographies (left panels) display the amplitude differences (µV) between inspiration and expiration separately for older adults. Electrodes belonging to significant clusters identified using cluster-based permutation tests (two-tailed t-tests) are marked by black squares, and stars indicate the significance level of the clusters (★★ *p* < 0.010, ★★★ *p* < 0.001). Numbers above each topography indicate the latency (ms) of the peak t-value within the significant cluster, with the cluster time window shown in parentheses. Grand-average ERPs averaged across electrodes belonging to the significant clusters are shown in the right panel (black = inspiration, red = expiration). Vertical dashed lines indicate that 0 s corresponds to target onset. Vertical shaded grey areas indicate the significant cluster time window.

Results for older adults are illustrated in Fig. 9. During the double-cue condition (Fig. 9a), older adults showed larger N1 amplitude during inspiration than expiration within 187–204 ms (*p* = 0.001) from target onset over right parieto-occipital areas. For no-cue stimuli (Fig. 9b), older adults showed larger N1 amplitudes during inspiration than expiration within 178–183 ms (*p* = 0.001) from target onset over left parieto-occipital areas. For incongruent stimuli (Fig. 9c), older adults showed larger P3 amplitude during inspiration than expiration in the left parieto-occipital areas within 290–300 ms (*p* = 0.003). For congruent stimuli (Fig. 9d), older adults showed larger P3 amplitudes during inspiration compared to expiration within 306–315 ms (*p* = 0.003) in the left parieto-occipital areas.

To directly test whether respiratory phase effects differed between age groups, cluster-based permutation unpaired t-tests were performed on the respiratory phase–difference waveforms (inspiration–expiration). These analyses revealed no statistically significant age-group differences in the magnitude or spatial distribution of N1 or P3 amplitudes for any ANT condition (p-value range: 0.084–1.000; t-value range: − 3.506–4.257). In summary, both young and older adults showed larger N1 responses to target stimuli during inspiration compared to expiration in the double-cue and no-cue conditions. Respiratory phase effects on the P3 component varied across task conditions and age groups at the descriptive level: young adults showed larger P3 amplitudes during expiration for incongruent targets, whereas older adults showed larger amplitudes during inspiration, while both age groups exhibited larger P3 amplitudes during inspiration for congruent targets. Importantly, direct statistical comparisons revealed no significant age-group differences in respiratory phase–related N1 or P3 effects.

### Cardiac cycle phase modulates visual N1 and P3 amplitudes similarly in young and older adults during ANT

**Fig. 10:**
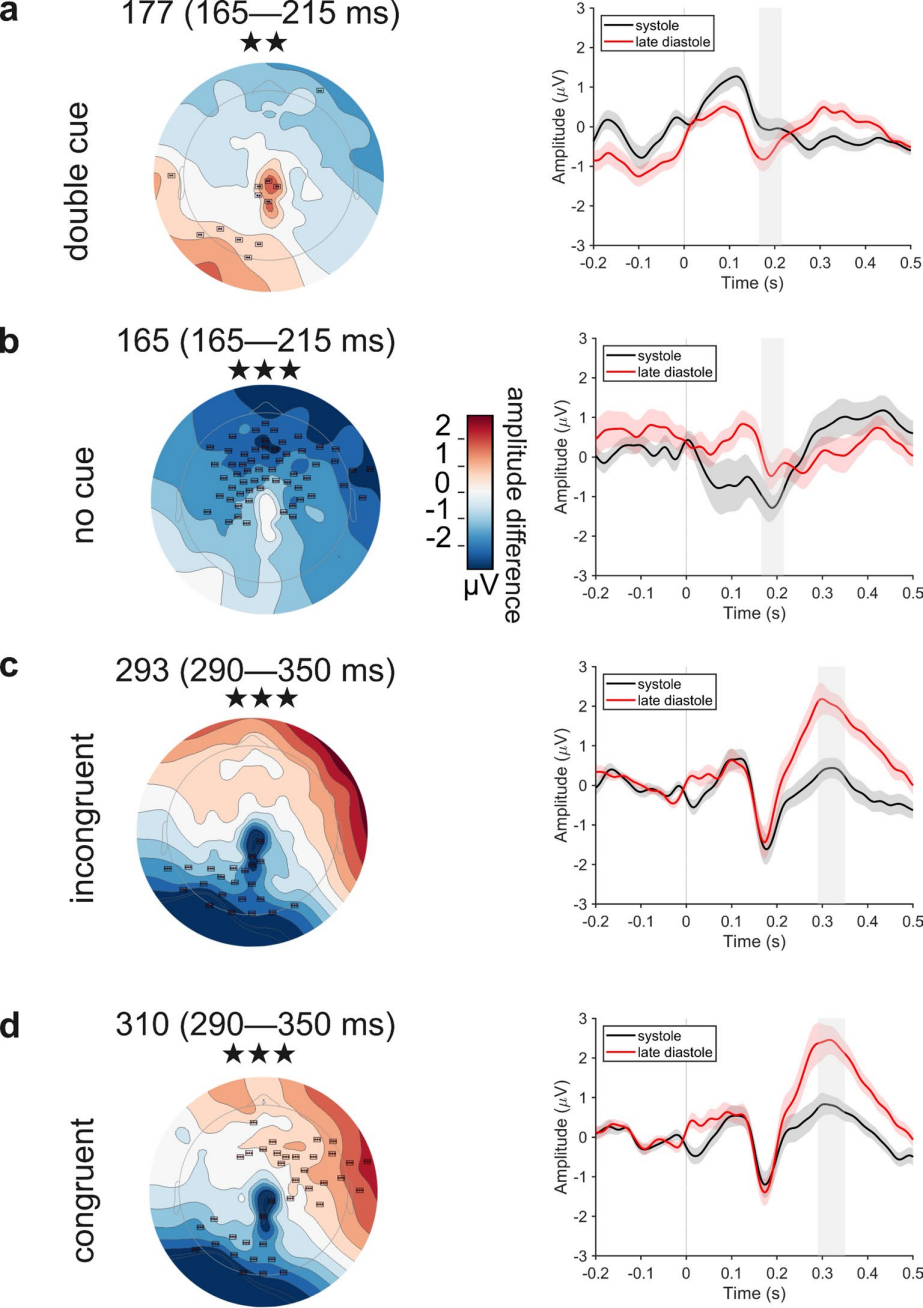
Topographical maps and event-related potentials (ERPs) showing the effects of cardiac phase (systole vs. late diastole) on visual target-evoked N1 (165–215 ms) and P3 (290–350 ms) amplitudes during the Attention Network Test in young adults. Rows correspond to stimulus conditions as follows: (**a**) double-cue, (**b**) no-cue, (**c**) incongruent, and (**d**) congruent. For each condition, scalp topographies (left panels) display the amplitude differences (µV) between systole and late diastole separately for young adults. Electrodes belonging to significant clusters identified using cluster-based permutation tests are marked by black squares, and stars indicate the significance level of the clusters (★★ *p* < 0.010, ★★★ *p* < 0.001). Numbers above each topography indicate the latency (ms) of the peak test statistic within the significant cluster, with the cluster time window shown in parentheses. Grand-average ERPs averaged across electrodes belonging to the significant clusters are shown in the middle panels for young and older adults separately (black = systole, red = late diastole). Vertical dashed lines indicate that 0 s corresponds to target onset. Vertical shaded grey areas indicate the significant cluster time window.

Results for young adults are illustrated in Fig. 10. Cluster-based permutation tests revealed that young adults showed significantly larger N1 amplitudes during late diastole than systole in the central and left occipital areas within 165–215 ms (*p* = 0.001) in response to double-cue targets (Fig. 10a). For no-cue stimuli (Fig. 10b), no significant differences in N1 amplitudes were observed between systole and late diastole in the parieto-occipital areas. However, there were statistically significant differences in the frontal area within 165–215 ms (*p* < 0.001) from the target onset, indicating larger N1 amplitudes during late diastole than systole.

For incongruent target stimuli (Fig. 10c), young adults exhibited significantly larger P3 amplitudes during late diastole than systole in the centro-parietal and occipital areas within 290–350 ms (*p* < 0.0001) from target onset. Also for congruent target stimuli (Fig. 10d), young adults had larger P3 amplitudes during late diastole than systole in the centro-parietal and occipital areas within 290–350 ms (*p* < 0.0001) from target onset.

**Fig. 11:**
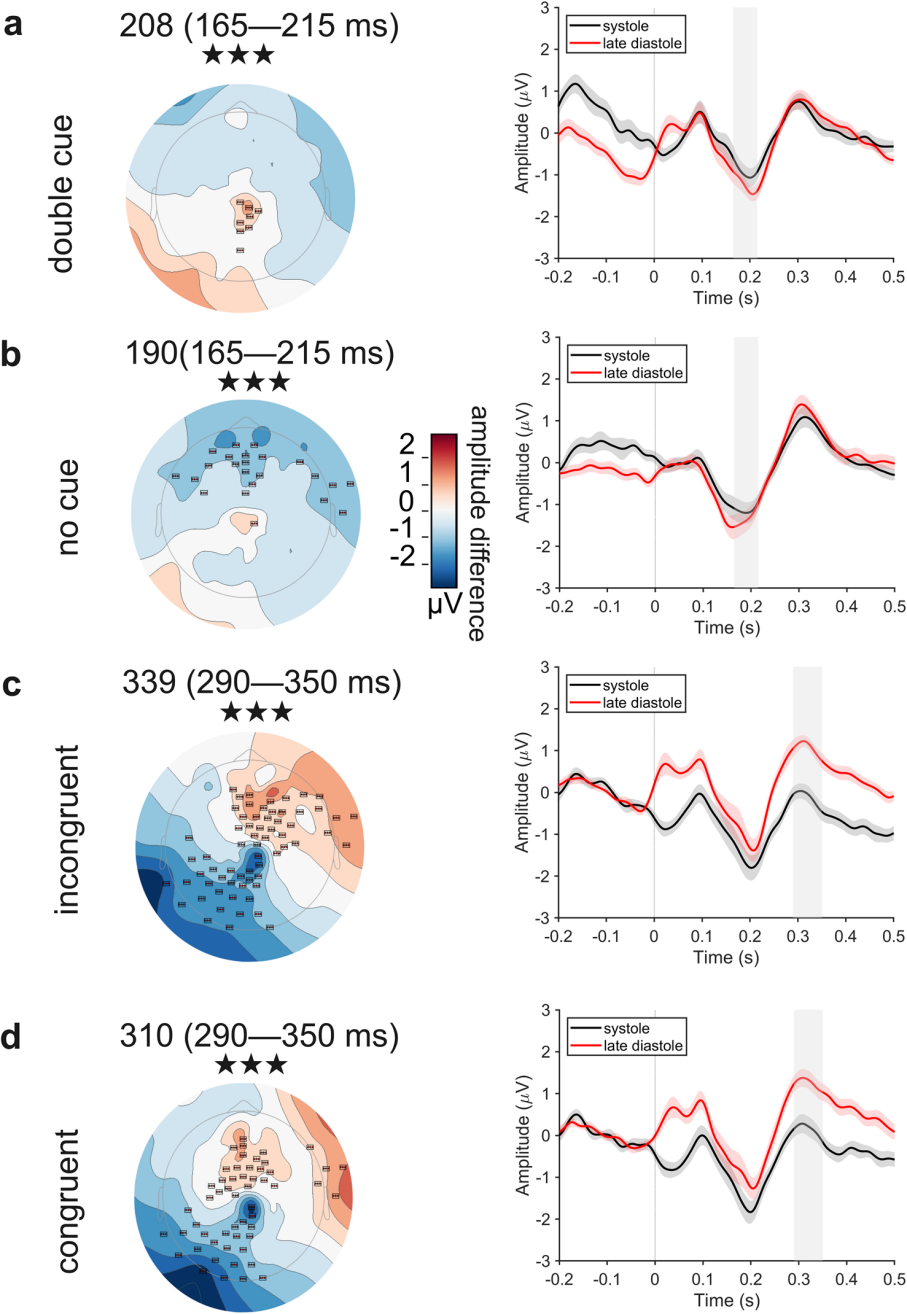
Topographical maps and event-related potentials (ERPs) showing the effects of cardiac phase (systole vs. late diastole) on visual target-evoked N1 (165–215 ms) and P3 (290–350 ms) amplitudes during the Attention Network Test in older adults. Rows correspond to stimulus conditions as follows: (**a**) double-cue, (**b**) no-cue, (**c**) incongruent, and (**d**) congruent. For each condition, scalp topographies (left panels) display the amplitude differences (µV) between systole and late diastole separately for older adults. Electrodes belonging to significant clusters identified using cluster-based permutation tests are marked by black squares, and stars indicate the significance level of the clusters (★★ *p* < 0.010, ★★★ *p* < 0.001). Numbers above each topography indicate the latency (ms) of the peak test statistic within the significant cluster, with the cluster time window shown in parentheses. Grand-average ERPs averaged across electrodes belonging to the significant clusters are shown in the middle panels for young and older adults separately (black = systole, red = late diastole). Vertical dashed lines indicate that 0 s corresponds to target onset. Vertical shaded grey areas indicate the significant cluster time window.

Results for older adults are illustrated in Fig. 11. Larger N1 amplitudes during late diastole than systole were observed in the centro-occipital area within 165–215 ms (*p* < 0.0001) in response to double-cue targets (Fig. 11a). For no-cue stimuli (Fig. 11b), no significant differences in N1 amplitudes were observed between systole and late diastole in the parieto-occipital areas; however, statistically significant differences were observed in the frontal area within 165–215 ms (*p* < 0.001) from the target onset, indicating larger N1 amplitudes during late diastole than systole. For incongruent target stimuli (Fig. 11c), older adults exhibited significantly larger P3 amplitudes during late diastole than systole in the centro-parietal and occipital areas within 290–350 ms (*p* < 0.0001) from target onset. For congruent target stimuli (Fig. 11d), older adults also showed larger P3 amplitudes during late diastole than systole in the centro-parietal and occipital areas within 290–350 ms (*p* < 0.0001) from target onset.

To directly test whether cardiac phase effects differed between age groups, cluster-based permutation unpaired t-tests were performed on the cardiac phase–difference waveforms (late diastole–systole). These analyses revealed no statistically significant age-group differences in the magnitude or spatial distribution of N1 or P3 amplitudes for any ANT condition (p-value range: 0.127–0.962; t-value range: − 2.847–3.193). In summary, both young and older adults showed significantly larger N1 and P3 amplitudes during late diastole compared to systole when responding to target stimuli across the different ANT conditions.

## Discussion

We investigated how phases of cardiorespiratory rhythms influence visual attentional subprocesses, alerting and conflict-related processing, in healthy young and older adults. Based on prior knowledge, we expected shorter RTs and larger brain responses to target stimuli presented during inspiration and diastole than during expiration and systole. Contrary to our expectation, RTs showed no reliable or systematic modulation by cardiac or respiratory phase and did not interact with age group or task condition. In contrast, brain electrophysiological responses revealed physiological modulation: respiratory phase produced task- and condition-specific effects on N1 and P3 amplitudes, whereas cardiac phase exerted a more global influence on these components, consistent with previous research^4,13,14,22^. Notably, the most robust finding of the present study was the global modulation of N1 and P3 amplitudes by cardiac cycle phase, which was consistent across task conditions and did not differ between age groups. At the behavioral level, young adults showed a larger alerting effect than older adults, while the conflict effect did not differ between groups. At the neural level, both age groups showed larger N1 and P3 amplitudes to double-cue and congruent target stimuli compared to no-cue and incongruent stimuli, respectively. Overall, young adults showed larger target-related brain responses than older adults, consistent with previous studies^18,19,23^. These findings are discussed in more detail below.

The behavioral results of our study indicate that both cue and target congruence significantly influenced RTs in both young and older adults. Cued and congruent target stimuli elicited faster responses than uncued and incongruent targets, respectively. These findings are consistent with previous studies showing that visual alerting cues enhance sensitivity to the incoming stimulus and thereby lead to faster responses^19,24–26^. Similarly, congruent target stimuli enable more efficient stimulus identification and response selection, contributing to faster responses^19,25^. In contrast, incongruent target stimuli slowed responses, reflecting increased demands for conflict monitoring and executive control, which are processes that engage executive control networks^24,27,28^. Importantly, we observed an age-related difference in alerting: older adults showed smaller RT benefits from cues than younger adults, suggesting reduced sensitivity to preparatory signals. This finding aligns with previous reports of general age-related slowing and diminished attentional readiness^19,20,23,29^. Executive control, however, did not differ significantly between age groups at the behavioral level, suggesting preserved response conflict resolution despite age-related changes in other attentional domains^18–20^. Notably, in older adults the behavioral benefit of cueing depended on target congruency, with larger cueing-related RT reductions for congruent than incongruent trials, whereas cueing and conflict effects were independent in young adults. This pattern remained after normalization of reaction times, indicating that the age-related difference in alerting was not solely attributable to general slowing.

In addition to RTs, alerting and conflict-related effects were also observed at the ERP level in both young and older adults. Specifically, we found larger N1 amplitudes for cued compared to uncued target stimuli over the occipital and parietal regions in both age groups. However, young adults showed significantly larger N1 amplitudes for cued targets than older adults, reflecting more effective visual processing of target stimulus properties^24,26,28^. Regarding the P3, both age groups in our study showed a P3 response associated with the processing of task-relevant and conflicting information^18,19,24^. However, older adults showed reduced P3 amplitude compared to younger adults. Such reductions in P3 amplitude are often interpreted as alterations in the ability to allocate attentional resources effectively, which may be associated with age-related decline in cognitive processing^19,30^. Regardless, older adults preserve attentional function possibly through compensatory mechanisms such as increased frontal recruitment^31,32^.

Our study revealed that respiratory phases (i.e., inspiration and expiration) modulate N1 amplitude in a task-dependent manner, with effects emerging selectively under specific attentional conditions rather than as a global state effect. Importantly, when averaged across all task conditions, respiration phase did not exert a global influence on N1 or P3 amplitudes, indicating that respiratory modulation of early visual processing depends on attentional context. N1 amplitudes were larger during inspiration than expiration and this effect was more pronounced in young adults. Our findings suggest that the inspiration phase enhances cortical excitability and facilitates early visual processing during attentional engagement. This interpretation is supported by previous studies showing that inspiration is associated with improved perceptual performance^3,5,33^ and enhanced cortical responsiveness, as reflected in increased early ERP amplitudes and alpha suppression^5,6,33^. These effects are thought to result from the rhythmic alignment between respiratory phase and cortical oscillations, which may optimize the timing of sensory input processing^34^. Supporting this view, visual detection performance has been shown to vary systematically across the respiratory cycle, with improved accuracy during inspiration compared to expiration^7^. Together, these findings indicate that respiratory influences on visual processing are primarily context dependent.

Our findings on P3 amplitude revealed distinct patterns during different respiratory phases in young and older adults. These respiratory phase effects on P3 amplitude were contingent on task demands and differed between congruent and incongruent conditions, consistent with the condition-specific nature of respiratory modulation. For congruent targets, both young and older adults exhibited larger P3 amplitudes during inspiration compared to expiration, suggesting enhanced stimulus evaluation and more efficient engagement of attentional resources^19^ during the inspiratory phase. Recent findings indicate that the respiratory phase can modulate higher-order cognitive functions, including conflict processing and decision-making^7^, which may underlie the observed P3 enhancement. In contrast, for incongruent targets, a different pattern emerged: young adults exhibited larger P3 amplitudes during expiration, while older adults showed larger P3 amplitudes during inspiration. In young adults, the expiratory phase may support conflict monitoring through increased sensorimotor engagement and a relaxed physiological state, as suggested by prior studies showing greater activation of sensorimotor networks during expiration^35^. In older adults, increased P3 amplitudes during inspiration may reflect a compensatory mechanism to enhance cognitive control under increased task demands, possibly due to age-related changes in cortical responsiveness or autonomic regulation^18,30^. Taken together, these findings highlight that the influence of respiratory phase on conflict-related executive control is shaped by both task context and age-related changes in neurophysiological functioning.

Consistent with the global ERP analysis, cardiac phase exerted a robust influence on both N1 and P3 amplitudes across task conditions. The influence of cardiac cycle on attentional subprocesses revealed increased N1 and P3 amplitudes during late diastole compared to systole across all ANT conditions in both young and older adults, except for the N1 amplitude in the no-cue condition. These modulations during late diastole align with the theory of interoceptive predictive coding, which posits that lower baroreceptor activity during this phase reduces internal signal interference, thereby improving the brain’s ability to process external stimuli^36–39^. The significant modulation of ERP amplitudes during diastole suggests that reduced baroreceptor activity may optimize cortical excitability and hence facilitate sensory perception^37^. However, the lack of N1 amplitude difference between systole and late diastole during the uncued targets in the parieto-occipital areas in both groups might reflect the absence of temporal expectation. This is consistent with prior studies showing that attention and expectation are essential for amplifying cardiac-phase influences on sensory processing^40^. Consistent with the previous finding, the increased P3 amplitude during late diastole may reflect a phase when reduced interoceptive signals allow for greater neural efficiency in processing the target stimuli^14^.

Despite task-specific respiratory effects, we did not observe robust age-related differences in the global modulation of N1 or P3 amplitudes by respiratory or cardiac phase. This suggests that the modulatory influence of these physiological rhythms on attentional processing is preserved across the age groups.

Our study has some limitations. We did not account for individual differences in cardiorespiratory function, such as lung capacity, which may influence attentional performance in both age groups. It would be valuable for future studies to investigate how individual factors like cardiorespiratory fitness, physical activity levels, body composition, or diet affect attentional subprocesses. In addition, both age groups included a higher proportion of female participants, which may limit the generalizability of the findings, particularly in the older group, given known sex-related differences in cardiovascular aging. Additionally, age-related shifts from nasal to more frequent oral breathing in older adults may alter airflow dynamics, respiratory effort, and sensory feedback, potentially impacting brain–body interactions. In subsequent studies, especially in older adults, the route of breathing should be more closely monitored during the experiment. There may also be individual differences in how ongoing endogenous or stimulus-evoked brain (oscillatory) activity aligns with cardiac and respiratory rhythms. In future studies, it might be useful to analyze data at the individual level, when possible. While ICA was applied to minimize physiological artifacts, residual cardiac signals and HEPs may have influenced EEG activity^41^. However, as the N1 and P3 were robust in our data, this should not have caused major distortion to our results.

In summary, our study demonstrates that cardiac cycle phase globally modulates attentional subprocesses indexed by N1 and P3 amplitudes, whereas respiratory phase influences these processes in a task- and context-dependent manner. Specifically, larger N1 amplitudes during inspiration under specific task conditions, and larger N1 and P3 amplitudes during late diastole compared to systole, indicate enhanced visual cue processing and sensory processing supporting response selection to target stimuli. Age-related differences in RTs and ERP amplitudes highlight the impact of aging on cognitive performance, although physiological phase effects were primarily evident at the neural rather than behavioral level. Together, these findings advance our understanding of how physiological rhythms interact with neural mechanisms of attention across the adult lifespan.

## Methods

### Participants and ethics statement

The study was conducted in compliance with the Declaration of Helsinki, and protocols were approved by the ethics committee of the University of Jyväskylä, Jyväskylä, Finland. Participants were recruited based on age and self-reported health status via email lists and public notice advertisements. We recruited 50 participants: 25 young (8 males, 17 females) aged 22 to 44 years (mean ± standard deviation: 29 ± 6 years) and 25 older (4 males, 21 females) aged 62 to 85 years (72 ± 5 years). Inclusion required participants to have no disabilities in hearing or vision, no history of psychiatric or neurological problems or head injuries, and no diagnosed memory impairment. None of the participants reported using medications known to affect brain function (e.g., antidepressants, benzodiazepines, or other psychoactive drugs). Forty-seven participants were right-handed, one was left-handed, and two were ambidextrous. Participants did not receive monetary compensation for taking part in the study. All participants provided written informed consent prior to the experiment.

### Physiological measures

Electroencephalogram (EEG) data were recorded using a high-density array of 128 Ag-AgCl electrodes in HydroCel Geodesic Sensor Nets (Electrical Geodesics Inc.). Electrode impedance was maintained below 50 kΩ, and data quality was continuously monitored throughout the measurement. EEG signals were online filtered between 0.16 and 250 Hz and referenced to Cz online. Three electrocardiogram (ECG) electrodes (Kendall, H92SG) were used to measure the cardiac cycle: one electrode was placed on the top of the right clavicle, one on the left lower ribs, and the third on the back of the neck for grounding. Respiration was recorded using a reusable fabric belt (RESPA00000, Spes Medica, Italy) fastened on the lower chest area/abdomen on top of clothes. All physiological data were recorded at a 1 kHz sampling rate with the NeurOne system (Bittium Biosignals Ltd., Finland).

### Attention Network Test (ANT)

Attention can be divided into subprocesses, orienting, alerting and executive control. The Attention Network Test (ANT) is a visual reaction time (RT) task designed to probe these attention subprocesses^24,25^. In this study, we focus on two of these subprocesses: alerting and executive control. Alerting is related to arousal and vigilance while executive control refers to the process of resolving conflict between the target and flanker, consistent with the ANT framework^24,42^.

Our version of the ANT (see Fig. 1) was constructed using the Presentation software (Version 20.2 Build 07.25.18, Neurobehavioral Systems, Inc., Berkeley, CA, USA). A black fixation cross was visible in the center of the white screen during the entire task. A fixation period of 400 to 1600 ms preceded the trial onset. In the double-cue trials, two asterisks were presented simultaneously above and below the fixation cross. The duration of the cue was 125 ms, which was followed by the fixation cross (375 ms) and then a single row of five horizontal arrows. The center arrow in the stimulus was the target and the two arrows on either side of the target are referred to as flankers. The stimulus array in each trial was presented either above or below the fixation cross at the same location where the double-cue appeared. For congruent trials, the flankers were in the same direction as the target and for incongruent trials the flankers were in the opposite direction relative to the target stimulus in the middle.

The participant sat approximately 110 cm from a 24-inch computer screen (resolution 1920 × 1080, refresh rate 60 Hz). The instruction was to look at the fixation cross and report the direction (left or right) of the middle arrow, the target stimulus, as quickly and accurately as possible by pressing the corresponding button (left or right) on the response box. Participants responded to the direction of the target arrow using their dominant hand. No specific instructions were provided regarding breathing; participants were allowed to breathe naturally and use their preferred breathing route (nasal or oral) during the task. The maximum duration of the target stimulus array in each trial was 1700 ms until a response was detected; thereafter, if there was no response it was considered an unattended trial and terminated. The maximum duration of each trial was 4000 ms.

A few practice trials, including all possible combinations, were conducted with each participant, providing feedback after each trial to ensure they understood the task. Next, two blocks of 288 randomized ANT trials were conducted. Each block consisted of all possible conditions in equal proportions: two cue conditions (no-cue and double-cue) and two target stimulus conditions (congruent and incongruent). Thus, altogether 576 trials were conducted. The entire experiment took ~ 40 min to complete. Between blocks, participants were offered water, candy, and a screen break of up to 5 min.

From the data obtained during ANT, alerting was assessed by comparing responses to target stimuli when preceded by the double-cue versus no-cue and executive control was analyzed by comparing responses to the target among incongruent vs. congruent flankers.

### Data analysis

#### Reaction times

RTs for each trial were measured from the onset of the target stimulus to the button press using MATLAB R2024b. Trials with unattended or incorrect responses were excluded from the mean RT calculation. Generally, aging can create larger differences in ANT RTs^20,43^. Therefore, we calculated a relative measure by dividing the RTs by the average RT for each participant and then multiplying by 100 to obtain values representing the percentage of mean. To assess the effect of cue presentation on RTs we calculated the size (%) of the alerting effect as follows: (no-cue RT - double-cue RT) / no-cue RT. Similarly, to assess the effect of the flanker direction on RTs the size of the conflict effect (%) was quantified as follows: (incongruent RT - congruent RT) / congruent RT.

#### Respiration and cardiac cycle phase

The respiratory phase in radians at any given time point was derived from the respiration signal using the Hilbert transform in MATLAB. Subsequently, the respiration cycle was divided into two bins: inspiration and expiration. The phase of the respiration cycle, which ranges from − π to π radians, was used to categorize the data. A cutoff point at 0 radians was applied, where negative values (ranging from − π to 0 radians) were classified as expiration and positive values (0 to π radians) as inspiration. We visually checked in all participants that the Hilbert-derived phase trace aligned with the respiration peak, such that the zero-radian crossing coincided with the peak of inspiration and − π/π radians corresponded to the end of expiration. Phase data were rounded to two decimal places to avoid binning errors caused by small numerical differences. Trials were sorted into inspiration and expiration bins based on the respiration phase at target onset. Across all participants, there were on average 528 ± 61 trials out of 576 with valid respiration data. Of these, 264 ± 40 trials took place during inspiration and 269 ± 36 during expiration. When further divided among the four ANT trial types (no-cue, double-cue, congruent, and incongruent), trial counts were comparable across conditions. For clarity, we report the mean number of trials per respiratory phase averaged across all four ANT conditions: 128 ± 4 trials during inspiration and 132 ± 4 during expiration, confirming that all conditions contained a sufficient number of valid trials for reliable ERP averaging.

MATLAB was also used to find R-peaks from the ECG signal. We computed the difference between the target onset time and the nearest R-peak preceding it. To determine the length (in ms) of the systolic and diastolic phases for each participant, intervals between adjacent R-peaks (inter-beat intervals, IBIs) were extracted from the ECG signal, and segments were created around each R-peak. The mean ECG waveform time-locked to the R-peak was then calculated. Based on the averaged ECG waveform, the QRS complex and subsequent relaxation phases were identified, and the cardiac cycle was divided into three bins relative to the R-peak: systole, early diastole, and late diastole. Following Al et al. (2020), these phases were defined proportionally relative to each participant’s cardiac cycle, with systole corresponding to 0–20%, early diastole to 21–70%, and late diastole to 71–100% of the inter-beat interval. These proportional boundaries were scaled to each participant’s mean IBI to account for individual differences in heart rate. On average, in young adults these phases corresponded to approximately 0–202 ms (systole), 202–593 ms (early diastole), and 593–907 ms (late diastole), whereas in older adults they corresponded to approximately 0–206 ms, 206–545 ms, and 545–946 ms, respectively. We divided the diastole into early and late phases to improve temporal precision^37,40^ and to detect potential phase-specific effects on attention. Finally, trials were sorted into the bins based on the time elapsed from the most recent R-peak to the target onset.

Across all participants, there were on average 527 ± 73 trials out of 576 with valid ECG data. Of these, 130 ± 25 trials took place during systole, 233 ± 59 during early diastole, and 170 ± 65 during late diastole. When further divided among the four ANT trial types (no-cue, double-cue, congruent, and incongruent), trial counts were comparable across conditions. For clarity, we report the mean number of trials per cardiac phase across all four ANT conditions (no-cue, double-cue, congruent, and incongruent): 65 ± 14 during systole, 116 ± 31 during early diastole, and 84 ± 33 during late diastole, confirming that all conditions contained a sufficient number of valid trials for reliable ERP averaging.

Furthermore, we evaluated whether scaling the bin boundaries using each participant’s IBI could lead to misclassification of cardiac phases (e.g., late-diastolic intervals extending into the next cycle). To assess this, we repeated the binning procedure using the same proportional boundaries (0–20%, 21–70%, 71–100%) applied to each individual R–R interval. Trial assignments from the two binning approaches showed very high concordance (93% in young adults and 94% in older adults), indicating that the results were robust to the choice of binning strategy.

#### Electroencephalogram

First, EEG signals were preprocessed using MATLAB R2022b, with FieldTrip^44^(version 20230427) toolbox. The continuous raw files were imported into MATLAB and bad channels were interpolated using the average method (FieldTrip). Eye blinks and cardiac artifact-related components (heart evoked potential, HEP)^45^ were removed using independent component analysis (ICA)^46^, excluding on average 2 cardiac-related and 2–3 ocular components per participant based on topography and time course. Although interpolation preceding ICA reduces the effective rank of the data, the number of interpolated channels was modest (mean = 3.92, SD = 2.32, range = 0–9 of 128 electrodes), making the impact on ICA decomposition negligible for the present analyses. Then, signals were bandpass-filtered between 1 and 30 Hz using a Hamming window. Data were segmented into epochs starting at 200 ms before the possible cue onset and ending at 500 ms after the target onset. This resulted in 1200-ms epochs. The baseline period was set at -200 to 0 ms relative to the possible cue onset. Trials with incorrect responses were excluded from further data analysis. Trials with muscle activity and other artifacts were excluded based on signal properties: If the difference between the maximum and minimum voltage in more than 25 electrodes exceeded 175 µV (or 300 µV in one electrode) that epoch was discarded. The accepted epochs were then re-referenced to the average of all electrodes to improve the signal-to-noise ratio.

Next, epochs were averaged for each participant and each condition to ensure data quality and to determine the time windows for analyzing N1 and P3 amplitude. The number of epochs used for averaging was 270 ± 22 in young adults (minimum 189) and 266 ± 31 in older adults (minimum 176). ERPs were visually inspected and compared to those of data from other participants to ensure data quality. Data from one young adult participant was excluded from further analysis because of excessive noise in the EEG signals. The parieto-occipital visual N1 (100–280 ms) amplitude is assumed to reflect visual processing of the target stimulus and is modulated by cue condition, and thus it was used as the neural measure of alerting^18,24^. Conflict-related brain processing was quantified by analyzing the modulation of the centro-parietal P3 (300–650 ms) amplitude evoked by the target following congruent vs. incongruent flankers^18,24^. To determine the target-related N1 and P3 latencies, we first obtained the grand-averaged ERPs across all participants for each condition. We then averaged the signals from the selected electrodes: The N1 response was calculated from Oz, O1, O2, PO7, PO8, POz, P3, and P4 while the P3 response was calculated from the Pz, POz, and Oz^18,24^. After inspecting the grand-average ERPs, the N1 time window was defined as 165–215 ms after target onset and the P3 time window was defined at 290–350 ms after target onset for both young and older adults.

### Statistical analysis

#### Reaction times

IBM SPSS Statistics version 28.0.1.1 (15) was used for the statistics. A mixed-design ANOVA with Cue (double vs. no-cue) and Conflict (congruent vs. incongruent) as within-subject factors and Age group (young vs. older) as a between-subject factor was used to examine effects on raw reaction times (ms). Any significant interactions were further examined with simpler ANOVAs. To account for overall slowing with age, RTs were also expressed as percentages of each participant’s mean (% of mean), and the same mixed-design ANOVA model was applied to these normalized RTs. In addition, independent-samples t-tests were used to compare the sizes of the alerting and conflict effects between age groups. For physiological phase effects, mixed-design repeated-measures ANOVAs were conducted to assess differences in relative RTs, with physiological phase (respiration phase or cardiac cycle phase) and task condition as within-subject factors and age group as a between-subject factor. Bonferroni correction was used in post hoc testing where applicable.

#### Event-related potentials

Non-parametric, cluster-based permutation tests were conducted using BESA Statistics 2.1 (BESA GmbH, Munich, Germany) to identify significant differences in ERP components N1 and P3 amplitudes across all electrodes within and between groups (young and older adults) for the two ANT effects: alerting (double-cue vs. no-cue target stimuli) and conflict-related processing (incongruent vs. congruent target stimuli).

For within-group comparisons, two-tailed, paired t-tests were used. For between-group comparisons, difference waveforms for each effect within each group were calculated using MATLAB and then imported into BESA Statistics 2.1 and then two-tailed, unpaired t-tests were performed. Based on the region of interest electrodes’ grand-averaged ERPs (see Fig. 6), the time window for cluster-based permutation tests was set between 165 and 215 ms after target onset for the alerting subprocess and between 290 and 350 ms after target onset for the conflict-related subprocess. The number of permutations was set to 1000, with a cluster alpha of 0.05 indicating the significance threshold for data inclusion in a cluster. Spatial clustering was achieved with a neighbor distance of 3.5 cm between electrodes. When multiple significant clusters were detected, the largest cluster is reported in the Result, whereas additional clusters are shown in the figures where relevant.

To assess differences in N1 and P3 amplitudes between the inspiration and expiration across different ANT conditions, we performed cluster-based permutation tests using BESA Statistics 2.1. For within-group comparisons, two-tailed, paired t-tests were conducted between the ERPs during inspiration and expiration phases during the N1 period (165–215 ms after target onset) and the P3 period (290–350 ms after target onset). For between-group comparisons, difference-waveforms representing the subtraction of ERPs during expiration from inspiration were calculated for each participant using MATLAB. These difference waveforms were then imported into BESA Statistics 2.1, where two-tailed, unpaired t-tests were performed to compare the groups.

Further, within-group comparisons were conducted using ANOVAs to assess differences in ERP N1 and P3 amplitudes between the cardiac cycle phases (systole, early diastole, and late diastole) across ANT conditions. However, the focus was on systole (i.e., peak cardiac contraction) and late diastole (i.e., peak cardiac relaxation), as these phases represent the most physiologically distinct points and are hypothesized to have an impact on brain activity and cognitive processes. Post-hoc paired t-tests were subsequently performed using BESA Statistics. For between-group comparisons of cardiac cycle phases, difference-waveforms representing the subtraction of ERPs during late diastole from systole were calculated for each participant using MATLAB. These difference waveforms were then imported into BESA Statistics 2.1, where two-tailed, unpaired t-tests were performed to compare the groups.

For visualization purposes, grand-average ERP waveforms shown in Figs. 7, 8, 9, 10 and 11 were extracted from cluster-derived regions of interest (ROIs), defined as the peak electrode and its neighboring electrodes belonging to each significant cluster. ERP waveforms were averaged across these electrodes within each participant and then across participants within each age group. In addition to the cluster-based permutation analyses, global effects of respiratory and cardiac phase on ERP amplitudes were assessed using repeated-measures ANOVAs. Subject-level mean N1 and P3 amplitudes were extracted from a priori defined regions of interest electrodes and (N1, P3) time windows and averaged across all ANT conditions. Respiration phase (inspiration, expiration) or cardiac phase (systole, early diastole, late diastole) was entered as a within-subject factor, and Age group (young, older) as a between-subject factor.

## Data Availability

The data that support the findings of the current article are available from the corresponding author upon reasonable request.
